# Real-Time Interaction between TBP and the TATA Box of the Human Triosephosphate Isomerase Gene Promoter in the Norm and Pathology

**Published:** 2014

**Authors:** O. V. Arkova, N. A. Kuznetsov, O. S. Fedorova, N. A. Kolchanov, L. K. Savinkova

**Affiliations:** Institute of Cytology and Genetics, Siberian Branch of the Russian Academy of Sciences, Lavrentyev Ave., 10, 630090, Novosibirsk, Russia; Institute of Chemical Biology and Fundamental Medicine, Siberian Branch of the Russian Academy of Sciences, Lavrentyev Ave., 8, 630090, Novosibirsk, Russia; Novosibirsk State University, Pirogov Str., 2, 630090, Novosibirsk, Russia

**Keywords:** TATA box, TBP, polymorphism, TBP/TATA-box interaction, stopped flow

## Abstract

The TATA-binding protein (TBP) is a key part of the transcription complex of RNA polymerase II. Alone or as a part of the basal transcription factor TFIID, TBP binds the TATA box located in the core region of the TATA-containing promoters of class II genes. Previously, we studied the effects of single nucleotide polymorphisms (SNPs) on TBP/TATA-box interactions using gel retardation assay. It was demonstrated that most SNPs in the TATA boxes of some human gene promoters cause a 2- to 4-fold decrease in TBP/TATA affinity, which is associated with an increased risk of hereditary diseases, such as β thalassemias of diverse severity, hemophilia B Leyden, myocardial infarction, thrombophlebitis, lung cancer, etc. In this work, the process of TBP/TATA complex formation has been studied in real time by a stopped-flow technique using recombinant human TBP and duplexes, which were identical to the TATA box of the wild-type and a SNP-containing triosephosphate isomerase gene promoter and were fluorescently labeled by the Cy3/Cy5 FRET pair. It has been demonstrated for the first time that real-time binding of TBP to the TATA box of the *TPI *gene promoter is complete within 10 s and is described by a single-stage kinetic model. The complex formation of TBP with the wild-type TATA box occurs 5.5 times faster and the complex dissociation occurs 31 times slower compared with the SNPcontaining TATA box. Within the first seconds of the interaction, TBP binds to and simultaneously bends the TATA box. Importantly, the TATA box of the wild-type *TPI *gene promoter requires lower TBP concentrations compared to the TATA box containing the -24T → G SNP, which is associated with neurological and muscular disorders, cardiomyopathy, and other diseases.

## INTRODUCTION

The specific nucleotide sequences of a promoter and around it serve as a code that determines when, where, and how efficiently certain genes are transcribed. This code consists of sequences of three types: the core promoter, the proximal promoter region, and distal sequences more remote from the promoter. The core promoter is a region situated at a distance of about 100 nucleotides upstream (in the 5’-region) and downstream (in the 3’-region) of the transcription start site, which comprises such regulatory elements as the TATA box, TFIIB-recognition element (BRE ), initiator (Inr), motif ten element (MTE ), downstream promoter element (DPE), downstream core element (DCE ), X core promoter element 1 (XCPE1), and others; their amounts may vary [1].

The TATA box, located at a distance of ~ 30 bps from the transcription start site, is the best-studied core-promoter element. Interaction between TBP (TATA-binding protein) and the TATA box initiates the assembly of the basal transcription complex of RN A polymerase II and determines the precision of the transcription machine location relative to the start nucleotide [1, 2]. The TATA box nucleotide sequence and the context in which it occurs determine its affinity for TBP, a subunit of the basal transcription factor, TFIID, which affects the promoter activity [3, 4].

Comparison of the TBP amino acid sequences of human, mouse, fruit flies, yeast, and other organisms has demonstrated that TBP is composed of the highly conserved C-terminal domain of 180 amino acid residues and a variable N-terminal domain [5]. The identity of the TBP C-terminal domain in different species is over 80% [5]. The X-ray analysis, footprinting analysis, and analysis of the location of the C-terminal domain tryptic peptides [6] revealed that TBP is composed of two subdomains, H2 and H2’, which form a continuous, slightly bent, antiparallel β-sheet, forming a concave DNA binding saddle, and of four α-helices that lie on the upper side of the molecule. The C-terminal domain of the TATA binding protein contacts the double- stranded DNA along the minor groove primarily through nonpolar and hydrophobic interactions and causes its local unwinding and helix bending. This creates a unique conformation that is crucial for the preinitiation complex assembly and efficient transcription both *in vitro *and *in vivo *[7]. Various regulatory proteins interact with the top, convex side of TBP [8].

Single nucleotide polymorphisms (SNPs) in TATA boxes and the surrounding nucleotides, which affect their affinity for TBP, can contribute to a variety of complex human diseases, such as hypertension, arthritis, cancer, cardiovascular and immune diseases. They can also cause monogenic diseases, such as β-thalassemias of varying severity, Coppock-like cataract, etc. [9].

The triosephosphate isomerase (*TPI*) gene is expressed in all cell types. It belongs to the housekeeping genes [10]. Multiple forms of TPI have been found in human tissues, which are encoded by a single gene and are formed as a result of posttranslational modifications [10]. TPI catalyzes the conversion of dihydroxyacetone phosphate to *D*-glyceraldehyde-3-phosphate, which completes the first step of glycolysis. A lack of the enzyme results in the accumulation of dihydroxyacetone phosphate and fructose diphosphate in the cell.

The -24T → G SNP in the TATA-box of the *TPI *gene promoter, reported in [11], leads to the synthesis of an insufficient amount of mRN A (hereinafter, under SNP is understood the G allele of the TATA box). The enzyme activity in erythrocytes of the allele carriers decreases and amounts to 3–10% of that in the cells of healthy donors [8, 11, 12]. They develop neurodegenerative disorders, cardiomyopathy, muscle disorders, and, less often, hemolytic anemia [11]. Furthermore, triosephosphate isomerase is capable of converting drug-resistant stomach cancer cells to sensitive ones [13], which improves the chemotherapy efficacy and makes the enzyme a potential target for new antitumor drugs.

Experimental and computational studies of the effect of SNPs within TATA boxes, which are in the context of the DNA of human gene promoters [14, 15], on the interaction with TBP has allowed us to determine the thermodynamic (*K*_D_) and kinetic (*k*_on_ and *k*_off_) parameters for the complex formation of TBP with the “normal” and SNP-containing TATA box of the *TPI* gene promoter.

Thus, it was demonstrated [14] that the -24T → G SNP in the TATA box of this gene strongly reduces the TBP/TATA affinity. The equilibrium dissociation constant of the complexes, *K*_D_, increases by 25 times, which correlates with the low gene expression [11]. In the presence of SNP, the rate constant of the TBP/TATA complex formation (*k*_on_) decreases by 35 times and the dissociation rate constant (*k*_off_) reduces by 30%.

The objective of the present work was to measure and analyze the kinetic parameters of the real-time TBP/ TATA interaction. The EMSA classical method, which was used to explore the thermodynamic and kinetic parameters of TBP/TATA complexes, does not allow for studying the interaction dynamics of TBP molecules and the TATA-box of the *TPI *gene promoter in the millisecond and second ranges. Therefore, binding of TBP to the TATA-box of the *TPI *gene promoter was studied using the “stopped-flow” method. The method is based on fast, within ~ 1 ms, mixing of the reactants and registration of the FRET (Forster Resonance Energy Transfer) signal. Recombinant full-length human TBP and 15 bp oligonucleotides identical to the TATA box with flanking nucleotides of the wild-type *TPI *promoter and the SNP-containing TATA box promoter and labeled with fluorescent Cy3 and Cy5 dyes were used in the study. This method enables one to determine the rate constant for the recognition of the wild-type TATA box by the TATA-binding protein and to reveal the structural features of the TBP/TATA complex in real time, under both normal and pathological conditions.

## EXPERIMENTAL

Only recombinant full-length human TBP containing the naturally occurring amino acid sequences was used in the study. TBP was expressed in BL21 (DE3)* Escherichia coli *cells transformed with the pAR3038- hTBP plasmid (kindly provided by Prof. B. Puhg, Center for Gene Regulation, Department of Biochemistry and Molecular Biology, Pennsylvania State University, University Park, PA, USA). BL21 (DE3) *E. coli *transformation was performed according to [16]. Expression and purification of TBP were performed according to the procedure described in [17] using the 0.1 mM IPTG concentration. The induction time was 3 h. A TBP concentration in a protein sample was determined by the Bradford method [18].

15 bp oligodeoxynucleotides (ODNs) labeled at the 5’-ends of the chains with cyanine fluorophores Cy3 and Cy5 were synthesized and purified at “NanoTekh- S”, Novosibirsk, Russia.

TBP binding to the DNA duplex, which corresponds to the wild-type TATA box of the *TPI *gene promoter (gctcTATA**T**AAgtgg, T allele, and gctcTATA**G**AAgtgg, G allele), was analyzed by the “stopped-flow” method on an SX20 spectrometer (Applied Photophysics, UK). The fluorescence excitation wavelength of the Cy3 dye was 550 nm; the voltage on the detector was 575 V. Cy5 fluorescence was recorded at wavelengths longer than 645 nm using the RG-645 filter (Scott, Germany). Binding to the DNA duplexes was studied using the following TBP concentrations: the T allele – 1 × 10^-7^, 2 × 10^-7^, 4 × 10^-7^, 6 × 10^-7^, 8 × 10^-7^, 10 × 10^-7^, and 20 × 10^-7^ M; the G allele – 4 × 10^-7^, 6 × 10^-7^, 8 × 10^-7^, 10 × 10^-7^, 20 × 10^-7^, 30 × 10^-7^, and 40 × 10^-7^ M. The DNA duplex concentration was 1 × 10^-7^ M in all cases, the measurement time was 50 s; the total number of points per curve was 6000. Experiments were conducted at 25°C.

To determine a kinetic model for the interaction of TBP with the DNA duplexes and to calculate the rate constants of all elementary reaction steps, the Dynafit software (Biokin, USA) [19] was used.

## RESULTS AND DISCUSSION

Studying pre-steady-state kinetics allows one to conduct a detailed analysis of the reaction mechanism. The advantage of the “stopped-flow” method is the opportunity it affords to observe transient reactions and to record the conformational transitions of a protein and DNA during a real-time interaction. Although this approach is technically more complex and its use requires a more labor-intensive mathematical analysis, studying the binding of the TATA binding protein to TATA boxes under pre-steady-state conditions enables one to deepen greatly knowledge about the mechanism of their interaction.

**Fig. 1:**
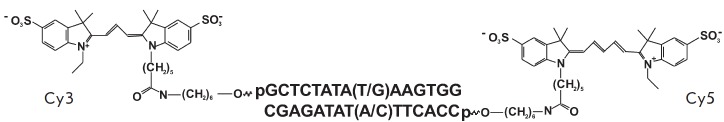
The DNA duplexes used were identical to the sequence of the TATA box of the wild-type (T/A) and the -24T → G SNP-containing *TPI *gene promoter and were labeled by the Cy3/Cy5 FRET pair

In this study, FRET substrates (*Fig. 1*) were used which contained a donor (Cy3)–acceptor (Cy5) pair at the duplex ends, while the central part of the duplex was the TATA box of the wild-type *TPI *gene promoter or that comprising the SNP.

**Fig. 2:**
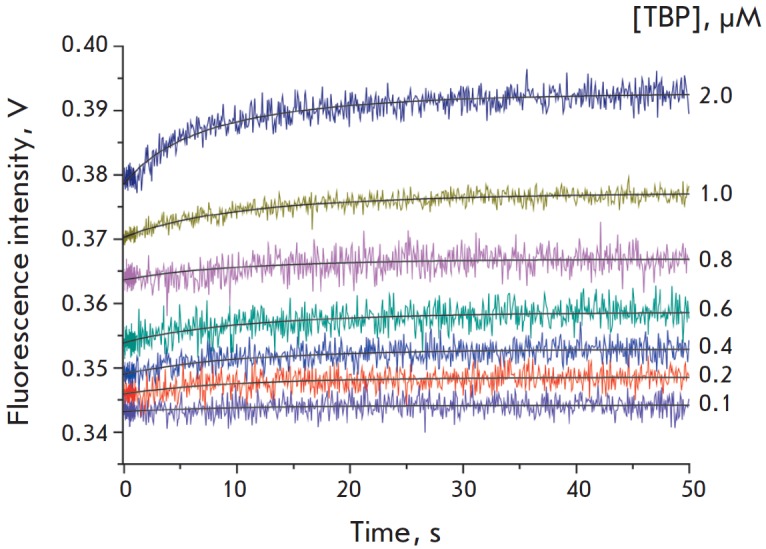
The kinetics of binding to and bending of the DNA duplex identical to the sequence of the TATA box of the wild-type *TPI *gene

**Fig. 3:**
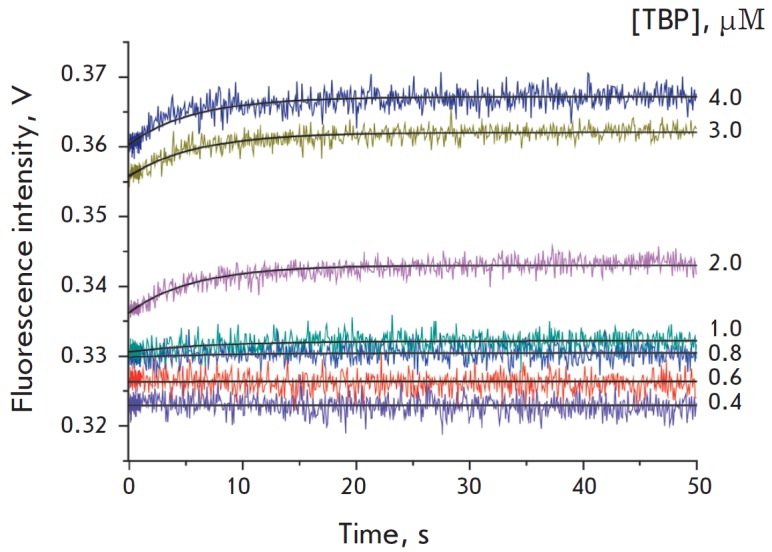
The kinetics of binding to and bending of the DNA duplex identical to the sequence of the SNP-containing TATA box of the *TPI *gene promoter

The kinetics of the binding of the DNA duplexes to TBP, presented in *Figs. 2* and *Fig. 3*, indicate that the TBP/ TATA complex formation leads to an increase in Cy5 fluorescence intensity. The increase in FRET signal intensity is caused by the bending of the DNA duplex in complex with TBP, which makes Cy3 and Cy5 fluorophore moieties approach one another. An analysis of the DNA duplex kinetic curves has revealed that the bending of the duplex containing the wild-type TATA box occurs at lower TBP concentrations than in the case of the G allele of the TATA box (*Fig. 2* and *Fig. 3*).

Based on these data, we have suggested a kinetic mechanism for TBP binding to the wild-type TATA box of the *TPI *gene and to the SNP-containing TATA box, which is described by a one-step *Scheme*:

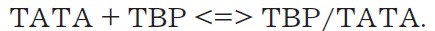



The rate constants for the forward and reverse reactions are given in *Table*. It is seen that the complex formation of TBP with the wild-type TATA box occurs 5.5 times faster (1.1 × 10^6^ M^-1^s^-1^) than with the G allele (0.2 × 10^6^ M^-1^s^-1^) and the dissociation of TBP/TATA complexes occurs 31 times slower (2.8 × 10^-3^ s^-1^ for the wild-type and 8.9 × 10^-2^ s^-1^ for the G allele). It should be noted that this difference in the rate constants of the TBP/TATA complex formation and decomposition leads to a difference in the values of the equilibrium dissociation constants by 150 times (2.7 × 10^-9^ M in the norm and 0.4 × 10^-6^ M in the presence of the mutation). The difference in the dissociation constant (*K*_D_) values between the wild-type and SNP-containing TATA box means a sharp decrease in the TBP affinity for oligonucleotides with an altered TATA box.

The obtained data indicate that the G/C-pair occurring in the TATA box makes the DNA structure more rigid, which complicates the TATA box binding to TBP and the formation of a functional complex possessing the optimal conformation. It implies that the triosephosphate isomerase gene containing the -24T → G SNP in the TATA box is *in vivo *transcribed and expressed less efficiently. These results have been confirmed clinically [11].

Comparison of our data and published ones [11, 20] demonstrates that the 150-fold decrease in the TBP affinity for the SNP-containing TATA box of the *TPI* promoter increases the risk of development of some diseases associated with the lack of triosephosphate isomerase. The lack of TPI may be compensated in other ways (e.g., in the pentose phosphate cycle), which follows from differences in the response of patients to TPI deficiency in the body [11, 21]. Despite the fact that TBP affinity for the SNP-containing TATA box of the* TPI *gene promoter is reduced 150-fold, TPI activity in the erythrocytes of some patients falls to 3–10% of the norm [21], and a moderate (26–50% of the norm) decrease in the TPI activity is observed in some heterozygous carriers of this polymorphic allele [11].

**Table:** The kinetics of interactions between TBP and the normal and the SNP-containing TPI TATA box

Constant	Normal TATA box	TATA box with -24T →G SNP
k_on_, M^-1^×s^-1^	(1.1 ± 0.1)×10^6^	(0.2 ± 0.1)×10^6^
k_off_, s^-1^	(2.8 ± 0.1)×10^-3^	(8.9 ± 1.2)×10^-2^
K_A_ M^-1^	3.7×10^8^	2.3×10^6^
K_D_, M	2.7×10^-9^ = 2.7 nM	0.4×10^-6^ = 400 nM

*Note*. *k*_on_ is the forward reaction rate constant for TBP/ TATA; *k*_off_ is the reverse reaction rate constant for TBP/TATA; *K*_A_ is the equilibrium association constant inferred from kinetic values (k_on_ / k_off_); *K*_D_ is the equilibrium dissociation constant inferred from kinetic values (k_off_ /k_on_).

It should be noted that by detecting the real-time interaction of human TBP with the Cy3 and Cy5 fluorescently labeled TATA-containing duplexes, it has been demonstrated for the first time that TBP rapidly binds to and simultaneously bends DNA of the* TPI *gene TATA box. This result is consistent with the data obtained previously using full-length human TBP and the AdMLP TATA box with a consensus sequence 5’-CGC**TATAAAA**GGGC-3’, the 5’-end of which was attached to the TAMRA fluorophore and the 3’-end was attached to fluorescein [22], which have indicated a one-step mechanism of the binding process and the simultaneous bending of the TATA box by TBP.

It should be noted that these studies have been conducted around the world using different TBP types, full and truncated forms (C-terminal domain), and primarily only the model AdML promoter (less often E4) with the TATA box consensus sequence. The obtained results improved the concept of the TBP/TATA interaction, which is the key interaction in the initiation and regulation of the transcription and synthesis of proteins in eukaryotic cells.

## Abbreviations

TBP: TATA-binding protein
TBP/TATA: complex of TBP with an oligonucleotide identical to the TATA box with flanking nucleotides
